# Forged by *DXZ4*, *FIRRE*, and *ICCE*: How Tandem Repeats Shape the Active and Inactive X Chromosome

**DOI:** 10.3389/fcell.2019.00328

**Published:** 2020-01-21

**Authors:** Prakhar Bansal, Yuvabharath Kondaveeti, Stefan F. Pinter

**Affiliations:** ^1^Department of Genetics and Genome Sciences, School of Medicine, UCONN Health, University of Connecticut, Farmington, CT, United States; ^2^Institute for Systems Genomics, University of Connecticut, Farmington, CT, United States

**Keywords:** tandem repeats, macrosatellite, X chromosome inactivation, chromosome conformation, chromatin loop extrusion, SMCHD1, XIST, intellectual disability

## Abstract

Recent efforts in mapping spatial genome organization have revealed three evocative and conserved structural features of the inactive X in female mammals. First, the chromosomal conformation of the inactive X reveals a loss of topologically associated domains (TADs) present on the active X. Second, the macrosatellite *DXZ4* emerges as a singular boundary that suppresses physical interactions between two large TAD-depleted “megadomains.” Third, *DXZ4* reaches across several megabases to form “superloops” with two other X-linked tandem repeats, *FIRRE* and *ICCE*, which also loop to each other. Although all three structural features are conserved across rodents and primates, deletion of mouse and human orthologs of *DXZ4* and *FIRRE* from the inactive X have revealed limited impact on X chromosome inactivation (XCI) and escape *in vitro.* In contrast, loss of *Xist* or SMCHD1 have been shown to impair TAD erasure and gene silencing on the inactive X. In this perspective, we summarize these results in the context of new research describing disruption of X-linked tandem repeats *in vivo*, and discuss their possible molecular roles through the lens of evolutionary conservation and clinical genetics. As a null hypothesis, we consider whether the conservation of some structural features on the inactive X may reflect selection for X-linked tandem repeats on account of necessary *cis*- and *trans*-regulatory roles they may play on the active X, rather than the inactive X. Additional hypotheses invoking a role for X-linked tandem repeats on X reactivation, for example in the germline or totipotency, remain to be assessed in multiple developmental models spanning mammalian evolution.

## Introduction

Since its initial discovery over 70 years ago (Barr and Bertram, 1949), the singular nature of the inactive X (Xi) in the female mammalian nucleus has captured the imagination of cell biologists studying chromosome organization, localization, and chromatin condensation. These studies have revealed the Xi to form the condensed “Barr body”, which localizes to the repressed nuclear periphery and periodically attaches to the nucleolus. The human metaphase Xi reflects this peripheral and peri-nucleolar localization with, respectively, alternating bands of tri-methylated lysines 9 and 27 of histone 3 (H3K9me3 and H3K27me3) (Vallot et al., 2016). During interphase, these chromatin domains of the human Xi segregate into compartments facing the nuclear interior or -lamina (Chadwick and Willard, 2004), and yet are hypothesized to mutually reinforce repression across this bi-compartmental Xi (Pinter, 2016). Recent studies have identified SMCHD1 as the principal *trans-*acting factor that bridges both H3K9me3 (H3K9me2 for the mouse Xi) – and H3K27me3-rich compartments to mediate *de novo* CpG island methylation for long-term Xi silencing, as reviewed recently (Jansz et al., 2017).

Rapid technical advances have enabled zooming into the unique three-dimensional (3D) topology of the Xi by chromosome conformation capture. As discussed below, these experiments have: (a) revealed how/when the Xi adopts its unusual chromosome conformation, (b) attributed the erasure of active X (Xa) topology to the concerted action of SMCHD1 and X chromosome inactivation (XCI) master-regulator *XIST/Xist*, and (c) implicated two conserved X-linked tandem repeats in shaping the Xi. Here, we review how these structural features relate to each other, and integrate findings from current *in vitro* and *in vivo* perturbation experiments with recent epigenomics and clinical genetics studies.

The central thesis of this perspective examines which of these Xi structural features may have been conserved due to important functions in XCI or escape, and which may emerge as mere “by-products” of the conserved Xi remodeling during XCI. These early results suggest that the *DXZ4/Dxz4* macrosatellite, while dispensable for XCI establishment, may have some limited impact on Xi choice. In contrast, accumulating evidence reveals that the *FIRRE/Firre* tandem repeat supplies critical *cis-* and *trans*-acting functions from its Xa allele. We therefore propose that *FIRRE/Firre* may have been conserved due to such XCI-independent roles. Alternatively, either or both of these conserved tandem repeats may participate in Xi biology in developmental contexts that have so far escaped analysis, for example in germline X reactivation or during zygotic genome activation.

## Unique Structural Features of the Inactive X

Eukaryotic chromosomes are composed of topologically associated domains (TADs) that consist of concentrated 3D interactions and organize into euchromatic “A” and heterochromatic “B” compartments (Nora et al., 2013). TADs are often bounded by convergent CTCF sites at the base of chromatin loops. These distal interactions are thought to result from loop-extruding DNA complexes that terminate at specific sites when movement of ring-shaped cohesin is blocked by architectural DNA binding factors like CTCF and YY1 (Rao et al., 2014; Sanborn et al., 2015). Not all cohesin loops constitute TAD boundaries, as intra-TAD loops enable long-range contacts between promoters and their regulatory elements (e.g., enhancers) (Dixon et al., 2016; Gonzalez-Sandoval and Gasser, 2016). Conversely, boundaries between A/B-type TADs (A/B boundaries) are also defined by local transitions in replication timing, lamin-association and chromatin composition, possibly due to intrinsic liquid-liquid phase-separating properties of lamin-associated B-type heterochromatin (Di Pierro et al., 2017; Schwarzer et al., 2017; Falk et al., 2019; Mirny et al., 2019). Since not all TAD boundaries coincide with cohesin loops, such A/B boundaries remain stable even when cohesin is depleted (Rao et al., 2017; Schwarzer et al., 2017).

### Cohesin Loop Erasure and TAD Attenuation

**FIGURE 1:**
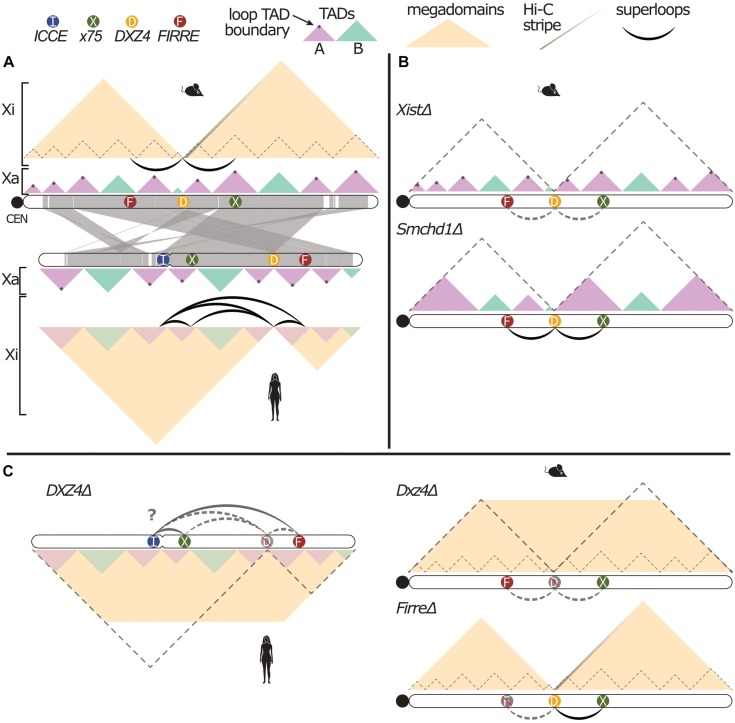
Conserved structural features of the mammalian inactive X. **(A)** Illustration of human and mouse X chromosome 3D conformation and synteny (generated with http://bioinfo.konkuk.ac.kr/synteny_portal/). A-type TADs (lavender) have cohesin loops demarcating boundaries of euchromatic TADs (as shown by the dark purple dots of increased Hi-C interaction), while B-type (aqua) TADs are largely heterochromatic. TADs observed on the Xa are lost (mouse) or attenuated (human) on the Xi, leaving two large megadomains. The stripe of contacts emanating from *Dxz4* on the mouse Xi represents the directionality of *Dxz4* toward the telomeric end of mouse X chromosome, and long-range superloops indicate 3D proximity of tandem repeats on both mouse and human Xi. **(B)** Mouse Xi conformation upon loss of *Xist* (top) or *Smchd1* (bottom). In *Xist*Δ, there is a loss of megadomains (dashed gray lines), re-established strength of the TADs (as represented by the increased opacity of the TADs), and a loss of superloops (as represented by the dashed gray lines). In *Smchd1*Δ, there is a loss of megadomains, and a merging of lavender A-type TADs. **(C)** Xi conformation after *DXZ4/Dxz4* or *Firre* deletions. Dashed gray lines represent loss of megadomains and superloops. Yellow trapezoid represents loss of contact isolation between megadomains. “D” or “F” are grayed and crossed out to indicate deletion of each superloop anchor. In both human (left) and mouse (right) *Dxz4*Δ, there is a loss of megadomain separation, Hi-C stripe and all superloops, with uncertain residual human superloops (“?”). In *Firre*Δ, there is only a loss of the *Dxz4-Firre* superloop.

The first structural feature to distinguish the Xi from the Xa (Figure 1A) may therefore be separated into two mechanistically distinct observations: (1) near-complete loss of long-range cohesin loops outside of escapee genes (Splinter et al., 2011; Nora et al., 2012; Rao et al., 2014), and (2) attenuation or loss of most TADs across the human and mouse Xi, respectively (Nora et al., 2012; Rao et al., 2014; Deng et al., 2015). Both of these observations have been conclusively linked to *Xist* RNA in the mouse: initial loss of TADs during XCI depends on *Xist*-mediated gene silencing, which facilitates spreading of *Xist* RNA into active genes (Engreitz et al., 2013; Chen C.-K. et al., 2016; Giorgetti et al., 2016). Because mouse XCI is maintained via stable CpG methylation, loss of *Xist* after completed XCI does not undo gene silencing, but allows chromosome topology to recover across the Xi to mirror the Xa conformation (Csankovszki et al., 1999, 2001; Splinter et al., 2011; Minajigi et al., 2015).

How does *Xist* RNA ablate both cohesin loops and cohesin-independent A/B boundaries across the Xi? In a seminal study, *Xist* RNA was shown to interact directly with cohesin subunits and reduce cohesin across the Xi (Minajigi et al., 2015). While the mechanistic basis for this loss remains unclear, *Xist* RNA effectively ablates cohesin loops that separate cohesin-dependent TAD boundaries, thus merging TADs (Figure 1B). In contrast, remodeling of cohesin-independent (largely A/B) boundaries depends on *Xist-*mediated recruitment of SMCHD1 (Wang et al., 2018) via polycomb-repressive complex 1 (PRC1) (Jansz et al., 2018; Gdula et al., 2019; Wang et al., 2019). In the absence of SMCHD1, *Xist* RNA is trapped in merged A-type TADs with persistent A/B boundaries (Wang et al., 2018). This observation is consistent with a previously described two-step model for how *Xist* RNA first spreads across gene-rich A-type TADs before entering LINE1/lamin-rich B-type TADs (Simon et al., 2013). Together, these reports suggest that SMCHD1 may dissolve cohesin-independent A/B boundaries by merging phase-separated, lamin-rich B-type TADs with *Xist/*PRC1-enriched A-type TADs. Supporting evidence for such hypothesized modulation of chromosomal phase-separation by *XIST/Xist* was recently summarized (Cerase et al., 2019).

### Megadomain Boundary: *DXZ4/Dxz4*

The second structural feature of the Xi emerges against the backdrop of this otherwise boundary-depleted chromosome conformation: the macrosatellite repeat *DXZ4/Dxz4* forms the only remaining topological boundary, thus separating the Xi into two large megadomains on the mouse, rhesus and human Xi (Rao et al., 2014; Deng et al., 2015; Minajigi et al., 2015; Darrow et al., 2016). While the *DXZ4/Dxz4* boundary is therefore conserved, gene content of the two megadomains is not evolutionarily fixed (Deng et al., 2015), as judged by mouse-human synteny maps (Figure 1A). Initially identified as a female-specific CpG-hypomethylated macrosatellite (Giacalone et al., 1992), a series of detailed studies by the Chadwick lab illuminated the enigmatic molecular configuration of this uniquely Xi-specific euchromatic region. On the Xa, transcription across both strands of its 3-kb long, CpG-rich repeat unit gives rise to small RNA transcripts that attract H3K9me3 heterochromatin and CpG-hypermethylation (Chadwick, 2008; Pohlers et al., 2014; Figueroa et al., 2015). In contrast, the CpG-hypomethylated Xi allele of *DXZ4* is decorated by active H3K4me3 and H3K9 acetylation marks that form a privileged euchromatic hub inside the otherwise repressed Xi chromosome territory. In human, rhesus and mouse, this euchromatic Xi allele of *DXZ4*/*Dxz4* binds CTCF and YY1 (McLaughlin and Chadwick, 2011; Horakova et al., 2012a, b; Moseley et al., 2012), and engages in Xi-specific long-range interactions. Notably, only the internal *DXZ4/Dxz4* promoter element containing paired CTCF and YY1 sites is conserved across mammals, suggesting possible selection for Xi-specific and CTCF-mediated functions in XCI across mammalian evolution (Horakova et al., 2012b; Westervelt and Chadwick, 2018).

Discovery of the Xi-specific megadomain boundary at *DXZ4/Dxz4* (Rao et al., 2014; Deng et al., 2015) prompted several groups to test whether its deletion impacted mouse or human XCI and escape (Figure 1C). Darrow et al. (2016) demonstrated that human *DXZ4* is required for boundary maintenance, and elegantly traced the 3D chromosome topology anchored at *DXZ4*, the *XIST-*proximal “*X75*” locus, and two distal tandem repeats, *FIRRE* and *ICCE*. Interestingly, in 2/3 RPE1 clones lacking *DXZ4* on the Xi, a quarter of cells replaced the largest H3K27me3 domain on the Xi with H3K9me3 (∼15 Mb adjacent to *DXZ4*), with a concomitant delay in replication timing. However, Xi-*DXZ4*Δ cells maintained XCI and the bi-compartmental interphase organization with nuclear interior-and lamina facing Xi domains of H3K27me3 and H3K9me3, respectively (Darrow et al., 2016).

These results were mirrored on the mouse Xi by Giorgetti et al. (2016): the megadomain boundary was lost in all four Xi-*Dxz4*Δ clones, with negligible impact on XCI establishment or maintenance in neuronal progenitor cells (NPCs) differentiated from mESCs. A single *Dxz4*Δ NPC clone featured reduced expression and chromatin accessibility of cell-type specific (facultative) escapee genes, along with a collapse of their residual TADs on the Xi. However, constitutive escapees remained unaffected, indicating that *Dxz4* is generally dispensable in mouse XCI and escape. Likewise, Froberg et al. (2018) found that *Dxz4*Δ abrogates the Xi megadomain boundary, but performed their experiments in the context of a *Tsix* mutation that rendered XCI non-random. This system enabled capturing the kinetics of megadomain boundary formation relative to *Xist* expression during XCI, while also assessing the impact of Xi-*Dxz4*Δ (-and *Firre*Δ) in differentiating mESC populations to exclude stochastic clonal phenomena. Indeed, the *Dxz4*-dependent megadomain boundary closely trails *Xist-*mediated gene silencing and TAD erasure, but chromatin accessibility and gene expression showed no significant changes in three independent *Dxz4*Δ clones, suggesting *Dxz4* is not required for XCI establishment and escape. Finally, Bonora et al. (2018) generated a series of edited *Dxz4* loci in immortalized Patski fibroblasts, separating its role from a proximal mouse-specific mini-satellite, DS-Tr. While *Dxz4* was indeed responsible for boundary maintenance, these editing experiments also produced two clones that inverted *Dxz4* and provided a critical mechanistic insight. As conserved CTCF sites in *Dxz4* share a common polarity, inverted *Dxz4* clones swapped the direction of 3D contacts anchored at *Dxz4*. Because CTCF sites block loop extrusion when paired in convergent fashion, the wildtype *Dxz4* boundary must therefore arrest cohesin loops extruded from the telomeric side of the Xi. These orientation-dependent Hi-C stripes emanate from *Dxz4* across ∼25–40 Mb of the Xi, indicating the remarkable persistence of cohesin rings traversing large swaths of the Xi (Figures 1A,C).

### Superloop Formation: A Euchromatic Hub of *DXZ4*, *FIRRE*, and *ICCE*

How does cohesin move past other convergent CTCF sites to extrude such extraordinarily long loops on the Xi? Although most CTCF peaks seen on the Xa are maintained on the human and mouse Xi (Calabrese et al., 2012; Ding et al., 2014; Kung et al., 2015), they appear attenuated on the Xi, especially in specific cellular contexts (Bonora et al., 2018). While the mechanistic underpinnings of this observation remain unclear, the presence or silencing function of *Xist* RNA (Kung et al., 2015; Minajigi et al., 2015) may impact CTCF residence time on DNA, e.g., via its RNA-binding domain (Hansen et al., 2017, 2019; Saldaña-Meyer et al., 2019). Cohesin rings may thereby be favored to traverse most CTCF sites on the Xi, until they encounter an array of stable CTCF sites at *Dxz4*, which is depleted of *Xist* RNA (Simon et al., 2013). A corresponding (paired) cohesin ring may therefore arrest anywhere along the telomeric stripe that originates at *Dxz4*, reflecting cohesin “dispersal”, across the Xi (Figure 1). This interpretation, as first proposed in Bonora et al. (2018), may also explain how a seemingly cohesin-depleted Xi (Minajigi et al., 2015) avoids premature sister chromatid separation to remain mitotically stable. Notably, except in cancer (Carone and Lawrence, 2013; Xu et al., 2017), the Xi has not been reported to suffer general mitotic instability even in the absence of *DXZ4/Dxz4*, suggesting that even dispersed cohesin rings maintain cohesion (Darrow et al., 2016; Giorgetti et al., 2016; Bonora et al., 2018; Froberg et al., 2018).

In the context of such cohesin “dispersal,” it appears perhaps unsurprising that the cohesin rings remaining on the Xi may eventually anchor at tandem arrays of stable and *Xist-*depleted CTCF sites that resemble *DXZ4/Dxz4*. As first reported by Horakova et al. (2012b) and later by Rao et al. (2014), *DXZ4* (at 115 Mb) is engaged in long-range, Xi-specific 3D contacts with two other X-linked repeats *FIRRE* (“*X130*”) and *ICCE* (“*X56*”), as well as the *XIST-*proximal “*X75*” locus (Figure 1A). Like the megadomain boundary, superloops between *DXZ4/Dxz4* and *FIRRE/Firre* are conserved in human, rhesus and mouse (Darrow et al., 2016). Although many possible pair-wise human Xi superloops were first observed (Rao et al., 2014), Darrow et al. (2016) demonstrated requisite engagement of *DXZ4* in most superloops by analysis of three-way proximity-ligated reads (COLA), and confirmed a co-localized *DXZ4-FIRRE-ICCE* hub at the single-cell level by FISH, as previously reported (Horakova et al., 2012b).

Like *DXZ4/Dxz4*, the *FIRRE/Firre* and *ICCE* tandem repeats reside inside Xa-transcribed genes (*FIRRE/Firre* and *NBDY*, respectively) and feature female-specific CpG hypo-methylation with paired CTCF-YY1 binding sites that are primarily occupied on the Xi (Ding et al., 2014; Hacisuleyman et al., 2014, 2016; Qu et al., 2015; Chen C. et al., 2016; Westervelt and Chadwick, 2018). *ICCE* is conserved across several mammals outside rodents, and likely derives from the ancestral *DXZ4* macrosatellite (Westervelt and Chadwick, 2018). On the mouse Xi, both *Dxz4* and *Firre* feature euchromatic H3K4me3 marks and are depleted of H3K27me3 and *Xist* RNA (Pinter et al., 2012; Simon et al., 2013), thereby sharing regulatory features of escapee genes despite residing inside genes that are subject to XCI (Berletch et al., 2010; Chen C. et al., 2016).

What is the function of this conserved euchromatic hub and does it depend on superloops? To address the latter question, Froberg et al. (2018) deleted *Firre* on the mouse Xi to remove the *Firre-Dxz4* superloop without disrupting the *Dxz4* anchor directly, demonstrating that the superloop is dispensable for *Dxz4* boundary function (Figure 1C). Moreover, neither *Dxz4*Δ, *Firre*Δ or double Xi-knockout cells reveal a consistent impact on XCI or escape. Likewise, female Xi-*Firre*Δ embryonic fibroblasts lose the *Firre-Dxz4* superloop, but maintain the syntenic mouse *x75-Dxz4* superloop (Barutcu et al., 2018). These data indicate that each Xi-linked superloop generally depends only on its own pair of anchors and the presence of the *DXZ4/Dxz4* hub. Yet, the role of this conserved euchromatic hub in Xi biology remains to be addressed, as do mutual dependencies of *DXZ4/Dxz4*, *FIRRE/Firre* and *ICCE* on CTCF binding and euchromatin maintenance.

## *Dissecting* Tandem Repeat DNA and RNA Functions *In Vivo*

While the results cited above focus on the Xi-linked roles of *DXZ4/Dxz4* and *FIRRE/Firre* in XCI *in vitro*, two new reports from the Rinn lab address their possible *in vivo* functions in development and XCI (Andergassen et al., 2019; Lewandowski et al., 2019). These studies touch on the critical question of whether there may be crucial XCI-specific functions of *DXZ4/Dxz4* or *FIRRE/Firre* that have been missed to-date, due to inaccessibility of certain developmental contexts with current mouse and human cell-based systems. However, discussion of these results necessitates drawing a distinction between *cis-*specific functions of these tandem repeats, and *trans*-acting roles of their RNA products.

While the *DXZ4/Dxz4* macrosatellite is thought to merely produce *cis-*acting short RNAs to maintain its heterochromatin on the Xa (Pohlers et al., 2014; Figueroa et al., 2015), the active *FIRRE/Firre* locus on the Xa also gives rise to multiple species of nuclear non-coding (nc)RNAs, which regulate autosomal genes, likely at the post-transcriptional level (Hacisuleyman et al., 2014, 2016; Bergmann et al., 2015; Izuogu et al., 2018). *Firre* RNA primarily regulates autosomal genes in the hematopoietic system, including in common lymphoid progenitors (Andergassen et al., 2019; Lewandowski et al., 2019). This observation may prove relevant to sex differences in autoimmune disorders (Syrett and Anguera, 2019), as the *FIRRE* locus was also recently identified as differentially methylated in CD4^+^ memory T cells of twins discordant for multiple sclerosis (Souren et al., 2019). While global *trans-*acting *FIRRE/Firre* RNA functions are well beyond the scope of this perspective, it appears that many autosomal targets function in RNA splicing, processing and transport, likely due to *FIRRE/Firre* RNAs physical association with HNRNPU (Hacisuleyman et al., 2014; Bergmann et al., 2015). This member of the large heterogeneous nuclear ribonucleoprotein family functions in mRNA splicing and processing, and has recently discovered roles in general genome architecture (Geuens et al., 2016; Zhang et al., 2019). Such roles may relate to the unusually stable *trans-*chromosomal hub anchored at *Firre* on the mouse Xa (Hacisuleyman et al., 2014). In addition, HNRNPU has fascinating but highly context-dependent roles in XCI (Hasegawa et al., 2010; Kolpa et al., 2016), as reviewed previously (Hasegawa and Nakagawa, 2011; Cerase et al., 2015; Pinter, 2016; Creamer and Lawrence, 2017).

Complicating the distinction from the CTCF-bound *Firre* locus on the Xi, predominantly Xa-transcribed *Firre* RNA was recently shown to play a role in tethering the Xi to the nucleolus via CTCF for maintenance of H3K27me3 (Yang et al., 2015). A new report from the Disteche Lab confirms that *Firre* cDNA expression rescues such H3K27me3 maintenance defects *in trans* (Fang et al., 2019). Interestingly, H3K27me3 dependency on *Firre* RNA appears to be confined to Patski and primary embryonic fibroblasts of the same interspecific cross (*Mus spretus* × *Mus musculus*), whereas H3K27me3-enrichment of the Xi in differentiating Xa-*Firre*Δ mESCs and primary tissues of Xa-*Firre*Δ mice appears unaffected in a pure *M. musculus* background (Yang et al., 2015; Fang et al., 2019). These differences between intra- and inter-specific F1 hybrids may be reconciled by global *trans-*acting changes encoded by the *M. spretus* genome. Such evolutionary divergence of *trans*-regulation (Signor and Nuzhdin, 2018) may therefore reveal a function for *Firre* RNA acting on the Xi. For example, compared with other mouse clades, the *M. spretus* genome appears to feature an overall reduction of stable CTCF binding (Kentepozidou et al., 2019), which may underpin the pronounced sensitivity to both CTCF and *Firre* RNA levels for nucleolar tethering of the Xi (Yang et al., 2015; Fang et al., 2019). Although the mechanism of this phenomenon remains to be explored, indeed CTCF binds *Firre* RNA (Graindorge et al., 2019), and CTCF peaks on the Xi are diminished in *Firre*Δ Patski cells (Fang et al., 2019). Another plausible explanation to reconcile differences between *Firre* RNA knockdowns and knockout mice/mESCs, could be that acute loss of *Firre* RNA affects H3K27me3 maintenance only on the Xi of differentiated cells, but may be compensated for if lost prior to initiation of XCI (Fang et al., 2019).

Yet, two new reports by the Rinn lab assess, but find no evidence for sex ratio distortion, or any *in vivo* defect in random or imprinted XCI establishment or maintenance in *Firre*Δ (Lewandowski et al., 2019), as well as single and double *Dxz4*Δ and *Firre*Δ mice (Andergassen et al., 2019). However, the latter study reports increased skewing of random XCI toward the *Dxz4*Δ Xi in these single- and double-knockout *Dxz4*Δ mice that merits follow-up. In sum, neither *Dxz4* nor *Firre* appear to be generally required for XCI establishment, maintenance or escape. Even double Xi knockouts of these tandem repeats, abolishing both megadomain boundary and superloops, appear to have little to no impact on XCI in mESCs (Froberg et al., 2018) and mice (Andergassen et al., 2019).

## *Firre* Duplication as a Potential X-Linked Intellectual Disability (XLID) Candidate?

If these X-linked tandem repeats are largely dispensable on the Xi, why were their sequence elements, chromatin composition and topology conserved across mammalian evolution? The *FIRRE/Firre* locus in particular illustrates how tandem repeats and macrosatellites compound special challenges intrinsic to molecular dissection of ncRNA loci (DNA vs. RNA/*cis-* vs. *trans*) (Bassett et al., 2014). Human (clinical) genetics studies can help link *FIRRE* to novel roles outside of XCI. These results have revealed increased CTCF binding and chromatin accessibility across the *FIRRE* tandem repeat in females (Ding et al., 2014; Qu et al., 2015), as well as differential CpG methylation in multiple sclerosis (Souren et al., 2019). In closing, we want to raise the question whether conservation of CTCF-mediated superloops on the Xi may be merely a consequence of evolutionary selection for critical regulatory functions originating from the Xa?

At present, there are two identified developmental roles that can serve as a basis for positive selection: (1) an established *trans-*acting function for *FIRRE* RNA in common lymphoid progenitors (Lewandowski et al., 2019), and (2) a hypothesized role in brain development, for which we refer readers to clinical reports of patients with copy-number gains in Xq26 (Schroer et al., 2012; Abe et al., 2014; Ha et al., 2019; Herriges et al., 2019). The latest of these reports summarizes sex-biased ID associated with duplications in this genomic region (Herriges et al., 2019), the shortest of which overlaps only *IGSF1*, olfactory receptor gene *OR1H*, and *FIRRE* (Abe et al., 2014).

**FIGURE 2:**
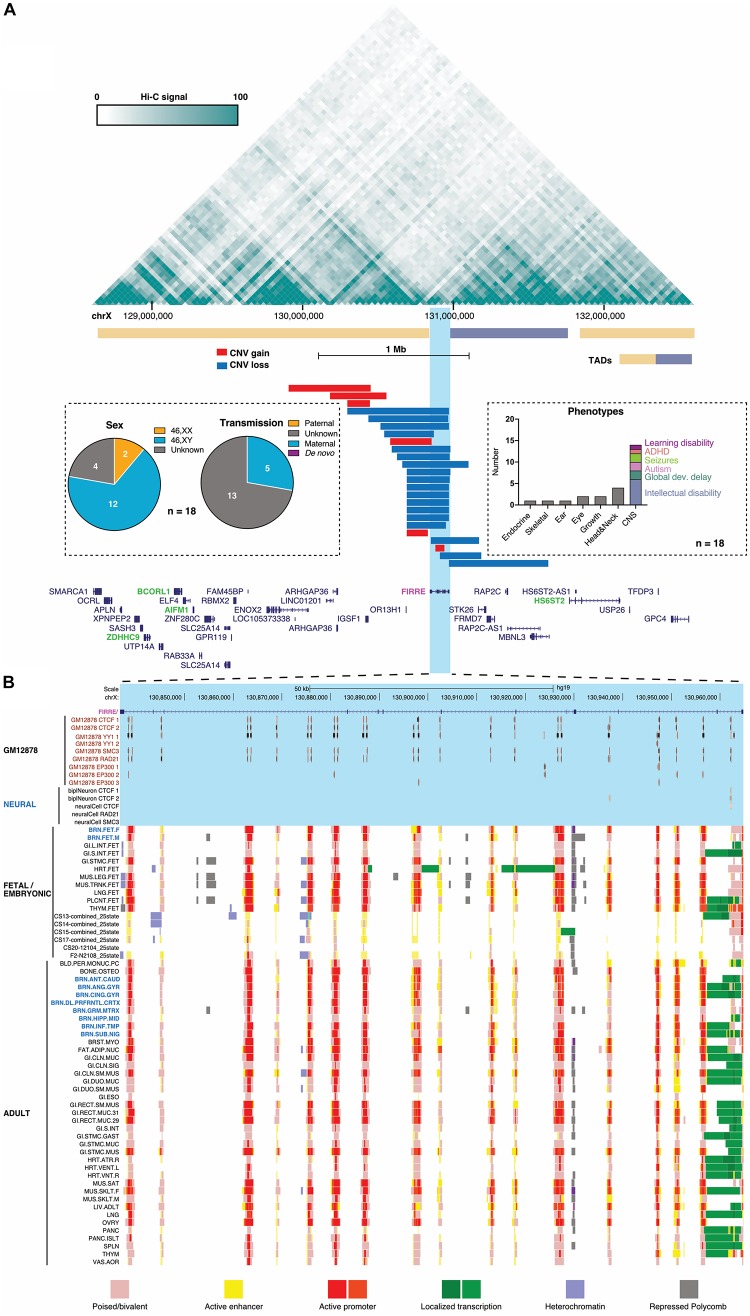
Conformation, copy-number variation (CNV) and chromatin signatures of the human *FIRRE* locus. **(A)** Prefrontal cortex Hi-C contact and TAD structure map from Schmitt et al. (2016) (top, generated with http://promoter.bx.psu.edu/hi-c/) of a 4-Mb region harboring FIRRE-overlapping CNVs in DECIPHER entries (gains blue, deletions red), in which no additional structural variation was detected. Names of genes located in the 4-Mb *FIRRE*-harboring region (bottom), with genes previously implicated in intellectual disability highlighted in green (OMIM #): *ZDHHC9* (300646), *BCORL1* (300688), *AIFM1* (310490), *HS6ST2* (300545). Insets tabulate the sex, transmission, and grouped clinical representations observed in DECIPHER patients carrying these *FIRRE-*overlapping gains. **(B)** Chromatin states and CTCF, YY1, cohesin, and P300 binding sites of the 130-kb human *FIRRE* gene. Poised/bivalent (Salmon), active enhancer (Yellow), heterochromatin (Pale Turquoise) or repressed polycomb (Silver) chromatin states form an array across *FIRRE* with localized transcription (Green/Lime Green) and active promoter activity (Red/Orange red). Data from across a variety of adult [Roadmap Epigenome (Yen and Kellis, 2015)] and developing human craniofacial (Wilderman et al., 2018) tissues.

Because additional cases may help to better resolve this region, we queried the DECIPHER database (Firth et al., 2009) and collated all short (<1 Mb) overlapping CNVs (Figure 2A). Tabulating phenotypes, transmission and sex only in patients who lack any other CNVs, these entries illustrate that: (1) almost all CNVs are gains that overlap *FIRRE*, (2) the associated phenotypes primarily involve the nervous system, and (3) most (12/14) patients are males who inherited the *FIRRE*-overlapping gain from weakly -or non-manifesting maternal carriers, where reported (Abe et al., 2014; Herriges et al., 2019). In contrast, among >5000 control individuals without pediatric disease in gnomAD-SV (Collins et al., 2019), 8/12 *FIRRE* duplications were present in heterozygous females, leaving one homozygous female and three males. While these low counts preclude strong conclusions at this time, and *FIRRE* duplications on the Xa are clearly not incompatible with neurotypical development, hemizygous *FIRRE* duplications appear to be over-represented in DECIPHER relative to this gnomAD-SV control cohort. Yet, certain biases in DECIPHER entries or an indirect contribution of *FIRRE’s* repeats to structural variation occurring in this region cannot be ruled out at this time.

If confirmed as a possible XLID risk locus, one interpretation of these data may suggest that *FIRRE* duplications, when present on the Xa, may tend to impact neurodevelopment: either by changing *FIRRE* RNA expression or *cis*-regulation of nearby genes by *FIRRE*’s array of CTCF sites. Importantly, *FIRRE*’s repetitive DNA elements were shown to confer enhancer activity *in vitro* (Hacisuleyman et al., 2016), contact neighboring genes in the human cortex (Schmitt et al., 2016) (Figure 2A), and attract a poised or active enhancer chromatin signature (Figure 2B) across a variety of adult (Yen and Kellis, 2015) and developing human tissues (Wilderman et al., 2018). Interestingly, the embryonic forebrain of *Firre*Δ mice shows significantly reduced expression of the neighboring *Hs6st2* gene (Lewandowski et al., 2019), the human ortholog of which was recently identified as a cause of X-linked ID in highly dosage-sensitive fashion (Paganini et al., 2019). Duplication-associated “re-wiring” of *FIRRE*-anchored promoter contacts may also increase or decrease expression of other neighboring genes, three of which are implicated in ID phenotypes in OMIM. All four of these XLID loci contact *FIRRE* in the developing human cortex (Figure 2). The compilation of these data are merely meant to caution against ruling out a potentially important and conserved *cis*-regulatory role for the *FIRRE* locus at this time (Lewandowski et al., 2019).

Whether the limited impact of *DXZ4/Dxz4* and *Firre* deletions on XCI *in vivo* (Andergassen et al., 2019) suggests an XCI-independent basis for conservation of these tandemly repeated CTCF/YY1 arrays, remains an open question for now. Alternative XCI-specific hypotheses may include *cis-*acting functions for X-linked tandem repeats in biological contexts that were not specifically explored by *in vitro* or *in vivo* experiments discussed here. In view of common chromatin features that *DXZ4*, *FIRRE*, and *ICCE* share with the DUX4-encoding *D4Z4* macrosatellite (Chadwick, 2009), we remain particularly curious about potential contributions of X-linked tandem repeats to human X reactivation phenomena in totipotency (Iturbide and Torres-Padilla, 2017), pluripotency (Geens and Chuva De Sousa Lopes, 2017), and primordial germ cell development (Payer, 2016).

## Data Availability Statement

All datasets generated and analyzed for this study are cited in the article/supplementary files.

## Author Contributions

PB and YK contributed equally and generated the figures. All authors wrote and edited the manuscript.

## Conflict of Interest

The authors declare that the research was conducted in the absence of any commercial or financial relationships that could be construed as a potential conflict of interest.
